# Dispersal of Transgenes through Maize Seed Systems in Mexico

**DOI:** 10.1371/journal.pone.0005734

**Published:** 2009-05-29

**Authors:** George A. Dyer, J. Antonio Serratos-Hernández, Hugo R. Perales, Paul Gepts, Alma Piñeyro-Nelson, Angeles Chávez, Noé Salinas-Arreortua, Antonio Yúnez-Naude, J. Edward Taylor, Elena R. Alvarez-Buylla

**Affiliations:** 1 Department of Agricultural and Resource Economics, University of California Davis, Davis, California, United States of America; 2 Universidad Autónoma de la Ciudad de México, México, Distrito Federal, México; 3 Departamento de Agroecología, El Colegio de la Frontera Sur, San Cristobal, Chiapas, México; 4 Department of Plant Sciences, University of California Davis, Davis, California, United States of America; 5 Laboratorio de Genética Molecular, Desarrollo y Evolución de Plantas, Instituto de Ecología, Universidad Nacional Autónoma de México, Distrito Federal, México; 6 El Colegio de México, Distrito Federal, México; 7 Universidad Autónoma Metropolitana, Distrito Federal, México; 8 Giannini Foundation of Agricultural Economics, Davis, California, United States of America; Cairo University, Egypt

## Abstract

**Objectives:**

Current models of transgene dispersal focus on gene flow via pollen while neglecting seed, a vital vehicle for gene flow in centers of crop origin and diversity. We analyze the dispersal of maize transgenes via seeds in Mexico, the crop's cradle.

**Methods:**

We use immunoassays (ELISA) to screen for the activity of recombinant proteins in a nationwide sample of farmer seed stocks. We estimate critical parameters of seed population dynamics using household survey data and combine these estimates with analytical results to examine presumed sources and mechanisms of dispersal.

**Results:**

Recombinant proteins Cry1Ab/Ac and CP4/EPSPS were found in 3.1% and 1.8% of samples, respectively. They are most abundant in southeast Mexico but also present in the west-central region. Diffusion of seed and grain imported from the United States might explain the frequency and distribution of transgenes in west-central Mexico but not in the southeast.

**Conclusions:**

Understanding the potential for transgene survival and dispersal should help design methods to regulate the diffusion of germplasm into local seed stocks. Further research is needed on the interactions between formal and informal seed systems and grain markets in centers of crop origin and diversification.

## Introduction

As increasing numbers of genetically modified crops are released into the environment, the likelihood of unintended ecological effects on both agricultural and natural systems increases. These effects become particularly relevant in centers of crop origin and diversity [1]. In Mexico, a country that harbors over 60% of maize's (*Zea mays* L.) genetic variation, gene flow among landrace and teosinte (wild *Z. mays*) populations has occurred readily since maize's domestication 9,000 years ago [2], [3]. But unlike domestication genes, which often represent a loss of function that decreases a plants' ability to survive without human intervention, many transgenes (*e.g.*, Cry genes) represent a gain of function that could enhance the survival or even the weediness of wild relatives [4], [5].

Assessing the potential for the dispersal of transgenes into crop landrace and wild populations is critical [6], [7]. The presence of transgenes in Mexican maize landraces was first reported in 2001 in the state of Oaxaca [8], but the extent of their dispersal is still in question. A subsequent study reported the presence of transgenes [9], while a third failed to detect them [10]. Some suggested that transgenes had disappeared, but recent studies have confirmed their presence in Oaxaca and found them in a new area of Mexico [11], [12]. Inconsistencies across studies might be due to differences in the analytical methods used or to narrow geographic sampling [12], [13]. Most analyses to date have been based on haphazard sampling of fields and seed stocks in a restricted number of localities; results are not representative of a well-defined population. Discrepancies might also be due to the dynamics of seed populations [13], [14]. However, the absence of proper data on seed dynamics and a formal framework to interpret these data has lead to widespread speculation.

In this paper, we analyze the implications of seed dynamics on the dispersal of maize transgenes across Mexico. There have been no commercial releases of genetically modified varieties (GMVs) of maize in Mexico, and there was a moratorium on all open-field plantings after 1998. However, seed of maize GMVs can be purchased in the United States (US), where it is widely planted, and brought into Mexico. US maize grain is another possible source of transgenes, since millions of tons of non-segregated grain have been imported and distributed throughout Mexican rural areas by the public retail network Diconsa. Seed and pollen exchange are both essential for the dispersal and persistence of alleles in cross-pollinated plants [15], yet there has been scant research on the effect of seed exchange on crop genetics [16], [17]. Current models of transgene dispersal focus almost exclusively on pollen exchange and the selective advantage of transgenes in wild populations [18]–[20]. Although they are well suited to industrialized agriculture, where seed is an input replaced every cropping cycle and seed exchange is absent, these models are not appropriate wherever seed is a capital asset saved across cropping cycles. In most centers of crop diversity, including Mexico, farmers save seed across cycles, forming local seed stocks, and they exchange seed among each other creating informal seed systems [6], [14], [21]. Seed systems consist of an interrelated set of components including breeding, management, replacement and distribution of seed [22]. In addition to seed systems, farmers occasionally use grain purchased as food or feed in lieu of seed [21]. Although there have been recent attempts to model the role of seed movement and anthropogenic factors in the establishment of feral crop populations and volunteers in industrialized agriculture [23], seed dynamics in centers of crop diversity constitute an entirely different phenomenon [6]. In contrast to pollen, which deposits largely within meters [18], [20], seed and grain can move thousands of kilometers, and seed replacement can alter local allele frequencies instantly and decisively [6], [16], [17]. Unsurprisingly, some analysts have assumed that maize germplasm introduced into Mexico, including GMVs, can diffuse rapidly across the country through informal seed systems and grain markets [24]–[26]. It is undeniable that genes can linger in or travel across local seed stocks as a result of farmers' decisions [6], [13], [15], but there are no quantitative analyses of this process. Here, we assess the potential for transgene dispersal via seed based on a model of crop populations and nationwide data on maize seed management [14]. We assess the distribution of transgenes across Mexico, and we test whether this distribution can be explained through different combinations of previously proposed mechanisms [9], [10], [13], [25].

## Materials and Methods

Studies of transgene dispersal face several methodological challenges. Although both polymerase chain reaction (PCR) and enzyme-linked immuno sorbent assays (ELISA) offer reasonable accuracy in the detection of transgene frequencies above 0.5%, frequency estimates themselves are still problematic [12], [27]–[29]. Quantitative estimates often depend on the screening method used [12], [28]. They also depend critically on the sampling framework [12], [27]. Even when transgene frequencies in a sample can be determined with reasonable accuracy, inferences on their frequency in the field must account for the structure and dynamics of the crop's metapopulation [12], [13]. Gene frequencies are scale-dependent due to the influence of population structure on gene flow. Spatial structure determines pollen exchange within and among individual plots in a locality during a single cropping cycle [19], [20], [30], but seed dynamics and management can have an overwhelming influence on the structure of populations across cycles and locations [14], [23], [30], [31]. It is misleading to estimate allele frequencies beyond the plot level without unraveling this complex population structure [12], [13].

In order to avoid these shortcomings, we focus here on the dynamics of relatively homogeneous populations, *i.e.*, seed lots, and the presence/absence of recombinant proteins within them. A seed lot is defined here as the set of kernels of a specific type (*e.g.*, shape, size or color) selected by a farmer and sown during a cropping cycle [30]. A transgenic seed lot is defined as one which contains one or more seeds expressing recombinant proteins. Seed lots and groups of seed lots that share some characteristic (*e.g.*, origin) are often subject to distinct rates of replacement and diffusion, which means that they constitute a well-defined seed population that can decrease or increase in numbers within the crop's metapopulation as a function of seed management [14]. Thus, the dispersal of genes within and across crop populations can be fostered or strictly limited by farmers' management practices.

The rate of growth (*λ*) of a closed seed population depends on the rates at which farmers save seed across cycles (*p*) and diffuse it (*q*) among a number (*C*) of fellow farmers: *λ* = *p*+*qC* [14]. In general, seed type *i* will grow as long as *λ_i_*>1. Seed that is not saved must be replaced, so that the rate of seed replacement is equal to *1−p*. In a metapopulation of constant size, a seed type that exhibits higher rates of replacement or lower rates of diffusion than the rest will decrease until it becomes extinct [14]. In 2002, the total maize acreage in Mexico was constant relative to previous years, and the estimated growth rate of the landrace metapopulation was *λ* = 1.03 [14]. Hence, differences in the rates of replacement and diffusion across maize seed types will indicate their propensity to spread within the metapopulation.

We estimated the frequency of presumed sources of maize transgenes and the rates of seed replacement and diffusion using data from the nationally representative 2002 Mexico Rural Household Survey (ENHRUM) [14]. This allowed us to analyze the presumed mechanisms of transgene dispersal into landrace populations. Using ELISA, we screened a collection of all maize seed types kept by survey households to determine the presence of transgenes. We tested for activity of two specific recombinant proteins from the most common commercial maize GMVs in the US in 2002: CP4/EPSPS (RoundUp Ready maize) and Cry1Ab/Ac (Bt maize). While PCR is perhaps the most common transgene detection method, ELISA's accuracy in qualitative analysis is comparable [27], [29]. ELISA has been thoroughly validated for transgene detection in maize [28], [29]; it offers clear advantages when screening large samples and is widely used in scientific research [11], [18], [32]–[34]. By screening directly for active recombinant proteins, we avoid technical problems associated with establishing the presence of recombinant DNA sequences from leaf tissue [12]. Our frequency estimates might be conservative if transgenes are present but inactive due to silencing [35] and given that we screen for only the most common recombinant proteins.

### Seed-lot sample and survey data

ENHRUM, the Mexico Rural Household Survey, was undertaken by the Programa de Estudios del Cambio Económico y la Sustentabilidad del Agro Mexicano, El Colegio de México, and the Rural Economies of the Americas Program, University of California, Davis, in collaboration with the Mexican census bureau (Instituto Nacional de Estadística, Geografía e Informática, INEGI). The survey is representative of the rural population nationwide and in each of the five regions in which INEGI divides the country. It is based on a stratified, three-stage cluster sampling frame designed by INEGI. Within each region, a sample of states, localities and households (*i.e.*, primary, secondary and elementary sample units, respectively) was selected through simple random sampling at every stage [36], [37]. Hence, our household sample consisted of 1765 households in 80 localities across 14 of the country's 31 states. The survey provides detailed information on the activities and assets of the rural population. ENHRUM also gathered data on every maize seed lot (*i.e.*, every distinct seed type) managed by households at the time of the survey, including detailed data for 2002 and retrospective data on seed diffusion for the previous 5 years. Data on 861 maize seed lots from 606 households were used to estimate rates of seed replacement and diffusion. Since these data are derived from a census of seed lots owned by surveyed households (*i.e.*, there was no sampling of seed lots within households), there are no sample design effects to consider other than those pertaining to the sampling of households themselves. As with most surveys, the precision of variance estimates derived from ENHRUM data is affected by its complex sample design. While clustering increases the variance of estimates, stratification entails a gain in precision of 21% relative to simple random sampling [36]. Although it is possible to correct for design effects on the variance of simple descriptive statistics (e.g., means and aggregates), no correction methods are available for most analytical statistics [36], [37], including the ones presented in this paper, which assume a simple random sampling of households. For a full discussion of ENHRUM's sample frame see <http://precesam.colmex.mx>. Rate differences were determined through the analysis of three-way tables based upon log-linear models [38].

### Seed sample and molecular analysis

Survey households also provided three seed-quality maize ears (*mazorcas buenas para semilla*) of every type they owned. This entailed selection out of seed stocks (or a harvest pile) according to farmers' criteria, which tends to sort out unintended crosses exposed by xenia when the pollen's genotype has a visible influence on the development of the endosperm [30]. A total of 419 seed lots were collected from 286 households in 49 localities across the 14 states. Seed replaced or discarded by households after the 2002 harvest was surveyed but not collected. Hence, the collection is representative of seed stocks at the beginning of 2003, which allows us to assess transgene dispersal up to the summer/fall 2002 cycle. Despite a larger sampling effort in the northeast and northwest, little maize was collected in those regions because commercial seed, which is common there, is replaced annually. The two regions are treated here as one. Seed is stored at El Colegio de la Frontera Sur (ECOSUR) and identified by blind-code collection numbers.

A wide number of transgenic maize events is available today and present in US grain exports [39], [40]. However, at the time of the collection, only three events expressing Cry1Ab/Ac and one expressing CP4/EPSPS had been deregulated and released commercially in the United States (according to information retrieved from the Agbios database <www.agbios.com> and the United States Regulatory Agencies Unified Biotechnology Website <http://usbiotechreg.nbii.gov/database_pub.asp>, accessed March 30^th^, 2009). Among the former, Bt11 and MON810 had been commercialized by 1997 and DBT418 (expressing Cry1Ac) by 2001. NK603, which expresses CP4/EPSPS, was commercialized in 2001. Another event expressing Cry1Ab/Ac (176) and three expressing both proteins (MON802, MON809 and MON80100) had been deregulated by 2002 but not released.

In order to maximize the number of pollination events sampled, two complete rows were removed from every ear in the ENHRUM collection and sown in bio-controlled greenhouse conditions until the six-leaf stage. Leaf tissue of 20 randomly-chosen individuals per ear was then pooled to integrate a single sample for each seed lot. Our protocol entails a sample size (n) of 60 seeds per lot, allowing detection of transgenic seed frequencies >0.045 (*i.e.*, >4.5%) at P<0.05 [12]. This corresponds to GMV seed lots and some advanced-generation seed mixtures resulting from different combinations of crossing; *e.g.*, selfing of GMV×non-GMV hybrid or backcrossing and reciprocals of a GMV×non-GMV cross with a non-GMV. In some cases, <60 seedlings per lot reached the six-leaf stage, reducing our ability to detect transgenes.

Commercial DAS-ELISA kits (Agdia, Elkhart, IN) used can detect 1 seed expressing CP4/EPSPS in 1000 and 1 leaf in 100 (www.agdia.com). A test of 1750 seed and leaf samples expressing CP4/EPSPS and 1750 conventional EPSPS samples, performed by the manufacturer, showed no false positives or negatives (www.agdia.com). We performed duplicate tests for each sample to increase the reliability of results [18]. In order to avoid contamination, tissue samples and controls were processed separately according to the standard protocol [11]. Kits were used on duplicate tests of 327 samples (10,979 individual seedlings) for CP4/EPSPS and 321 samples (10,679 seedlings) for Cry1Ab/Ac. As a negative control for both assays, we used leaf tissue of glufosinate resistant maize from the biolistic transformation of the CML72×CML216 hybrid introducing the *pat* gene (encoding phosphinothricin-N-acetyltransferase). CML72 and CML216 are two of CIMMYT's tropical inbred maize lines. Leaves of maize plants expressing Cry1Ab/Ac and CP4/EPSPS were used as positive controls [41]. Optical density (OD) was measured at 650 nm in a spectrophotometer after incubating for 10 minutes. Positive controls showed readings equal to the positive lyophilized protein provided with the kit. Negative controls (CML72, CML216) were consistently non-reactive to CP4/EPSPS and Cry1Ab/1Ac. Positive threshold values (*Th*) were defined as OD mean+5 SD of the normalized blank and negative control leaf tissue values, which is a more stringent criterion than the manufacturer's. Thresholds were set to *Th_CP4_* = 0.154 for CP4/EPSPS and *Th_Cry_* = 0.142 for Cry1Ab/Ac. Only samples with duplicate positive measurements (above the threshold) were considered positive. Analytical results were used to estimate frequencies of seed lots containing transgenes at the regional and national level but not the frequencies of transgenes within seed lots. A focus on presence/absence of transgenes at the seed lot level is entirely compatible with our interest in long-distance dispersal via seed. We have analyzed transgene dispersal at the locality level using a very different methodology and report our findings elsewhere [see ref. 12].

## Results

### Seed management and dynamics

According to ENHRUM data, between 1997 and 2001, 0.5% of Mexican rural farmers sowed maize seed brought from the US, but none of them conserved this seed in 2002 (Table 1). Nearly 3% of farmers sowed maize grain obtained in Diconsa, the public retail network, at least once during the same 5-year period, but only 0.5% of seed lots sown in 2002 came from this source. Seed obtained from government agencies was nearly as common as Diconsa's, while the formal seed system and other sources of grain each account for 10 times more seed. Seed exchange with other farmers through informal seed systems was overwhelmingly the main source of seed across Mexico (Table 1). Its importance is much greater in the southeast than in the north, where the seed industry and other institutional sources are also significant.

**Table 1:** Percentage distribution of original sources of maize seed across regions in Mexico in 2002.

Region	No. of seed lots	Seed sources (%)						Grain sources (%)	
		Other farmers^1^	Farmers' markets^2^	Government^3^	Other institutions^4^	Seed industry^5^	Foreign source* 6	Diconsa grain*	Other grain^8^
National	736	85.5	0.1	0.4	3.4	5.2	0.0 (0.0–0.3)	0.5 (0.2–1.5)	4.9
Southeast	266	95.5	0.0	0.4	0.4	0.8	0.0 (0.0–0.7)	0.0 (0.0–0.7)	3.0
Center	282	92.9	0.4	0.4	1.8	2.1	0.0 (0.0–0.7)	0.0 (0.0–0.7)	2.5
West-Center	111	64.0	0.0	1.8	3.6	17.1	0.0 (0.0–1.7)	1.8 (0.8–7.2)	11.7
North	77	54.5	0.0	0.0	18.2	14.3	0.0 (0.0–2.5)	2.6 (1.5–11.2)	10.4

* Confidence intervals (in parentheses) were estimated using profile-likelihood and binomial ln(-ln) transformations.

1 Friends, neighbors and relatives.

2 Farmers who sell seed openly to the public.

3 Government agencies and programs, e.g., Kilo por Kilo.

4 Intermediaries, private firms and banks.

5 Private seed companies.

6 Any source outside of Mexico.

7 Any source of grain other than Diconsa.

Analysis of seed replacement rates through separate goodness-of-fit tests revealed differences based on the type (P<0.001) and location (P<0.001) of seed sources (Table 2). Seed introduced into a locality and seed obtained through the formal system were replaced most often. A log-linear model was used to test for interactions of source type and location effects [38]. Only seed obtained through informal systems or as grain was included in this model, since all commercial seed is introduced, by definition. G-tests revealed significant interactions of replacement rates with source type (P = 0.002) and location (P<0.001) (Table 2). Freeman-Tukey deviates showed that seed obtained from neighbors was less likely to be replaced than seed from farmers outside the locality (*i.e.*, introduced seed) or seed grain acquired locally; but seed from all non-local sources was replaced at the same rate. Separate log-linear models controlling for the locality's altitude confirmed the effect of source type and location (P<0.001) while evincing marginally significant altitudinal effects (P = 0.10) (Table 3). Introduced seed is replaced more in low altitudes; local seed is replaced less in high altitudes.

**Table 2:** Source effects on rates of maize seed-lot replacement (*1−p*) and diffusion (*q*) in Mexico^1^.

Seed source	Replacement by source location^2^ (N = 716)			Diffusion by ownership^3^ (N = 711)			Diffusion by source location^2^ (N = 711)		
	local	Introduced	total	own	new	total	local	introduced	total
Informal system	0.18	0.54	0.21	0.24	0.18	0.22	0.23	0.20	0.22
Grain seed	0.70	0.55	0.63	0.13	0.12	0.13	0.15	0.10	0.13
Formal system	—	0.93	0.93	0.00	0.03	0.02	—	0.03	0.02
Total	0.19	0.69	0.27	0.23	0.15	0.21	0.22	0.12	0.21
G source effect	12.5** (2 df)			0.9 (2 df)			0.9 (2 df)		
G origin/ownership effect	15.6** (2 df)			1.0 (2 df)			0.2 (2 df)		

Significant at the 0.05 level is indicated by **. G-tests exclude seed from formal seed systems.

1 Expressed as a ratio, rates vary between 0 and 1. Replacement implies that seed is not saved by a farmer across cycles; diffusion entails the exchange of saved seed among farmers.

2 The terms “local” and “introduced” refer to the origin of the immediate source of seed; e.g., seed is local if acquired from neighbors, while seed acquired from farmers in another locality is introduced.

3 Seed acquired during the current cycle is “new;” seed saved by the farmer from a previous cycle is his/her “own.”

**Table 3:** Altitude and source effects on rates of maize seed-lot replacement (*1−p*) and diffusion (*q*) in Mexico^1^.

Altitude	Replacement by source location^2^ (N = 744)			Replacement by source type^2^ (N = 744)				Diffusion by source location^2^ (N = 739)			Diffusion by ownership^3^ (N = 739)		
	local	introduced	total	informal	grain	formal	total	local	Introduced	total	own	new	total
Low (<1200masl)	0.24	0.81	0.31	0.24	0.56	1.00	0.31	0.22	0.08	0.21	0.23	0.17	0.21
Mid (1200–2000masl)	0.21	0.62	0.36	0.25	0.58	0.91	0.36	0.19	0.09	0.15	0.19	0.10	0.15
High (>2000masl)	0.17	0.67	0.23	0.20	0.80	0.83	0.23	0.22	0.21	0.22	0.25	0.14	0.22
Total	0.20	0.67	0.28	0.22	0.63	0.93	0.28	0.22	0.13	0.20	0.23	0.14	0.20
G source/ownership effects	105.5** (3 df)			28.8** (3 df)				6.2* (3 df)			7.7** (3 df)		
G altitude effect	7.72* (4 df)			3.54 (3 df)				4.5 (4 df)			3.1 (4 df)		

Significant at the 0.05 level is indicated by **; significance at the 0.10 level is indicated by *. G-tests exclude seed from formal seed systems; masl: meters above sea level.

1 Expressed as a ratio, rates vary between 0 and 1. Replacement implies that seed is not saved by a farmer across cycles; diffusion entails the exchange of saved seed among farmers.

2 The terms “local” and “introduced” refer to the location of the immediate source of seed; e.g., seed is local if acquired from neighbors, while seed acquired from farmers in another locality is introduced.

3 Seed acquired during the current cycle is “new;” seed saved by the farmer from a previous cycle is his/her “own.”

Goodness-of-fit tests revealed differences in diffusion rates based on seed source (P = 0.003) and source location (P = 0.01) as well as on whether seed was newly acquired or saved (P = 0.01) (Table 2). Introduced, newly-acquired and commercial seed were diffused the least. Differences were largely restricted to introduced commercial seed, which was mostly newly acquired. Although no significant interaction effects were found in the diffusion of seed obtained through informal systems and as grain, complete independence of diffusion rates on source type and source location (P = 0.70; G = 1.4, 3df) and on ownership (P = 0.53; G = 2.2, 3df) could not be rejected when seed from formal systems was excluded from the analyses (Table 2). In separate tests controlling for altitude, marginally significant source location (P = 0.10) and ownership (P = 0.05) effects were evident, but no altitudinal effects on diffusion rates were found (P = 0.34, 0.53) (Table 3). As we have said, rate differences among seed types show that some populations spread within the metapopulation (*e.g.*, landraces acquired from neighbors) while others contract (*e.g.*, introduced seed and grain). Differences also allow us to trace the likely fate of germplasm as seed travels across categories (*e.g.*, after newly introduced seed is saved and incorporated into local stocks).

### Detection of transgenes

**Figure 1:**
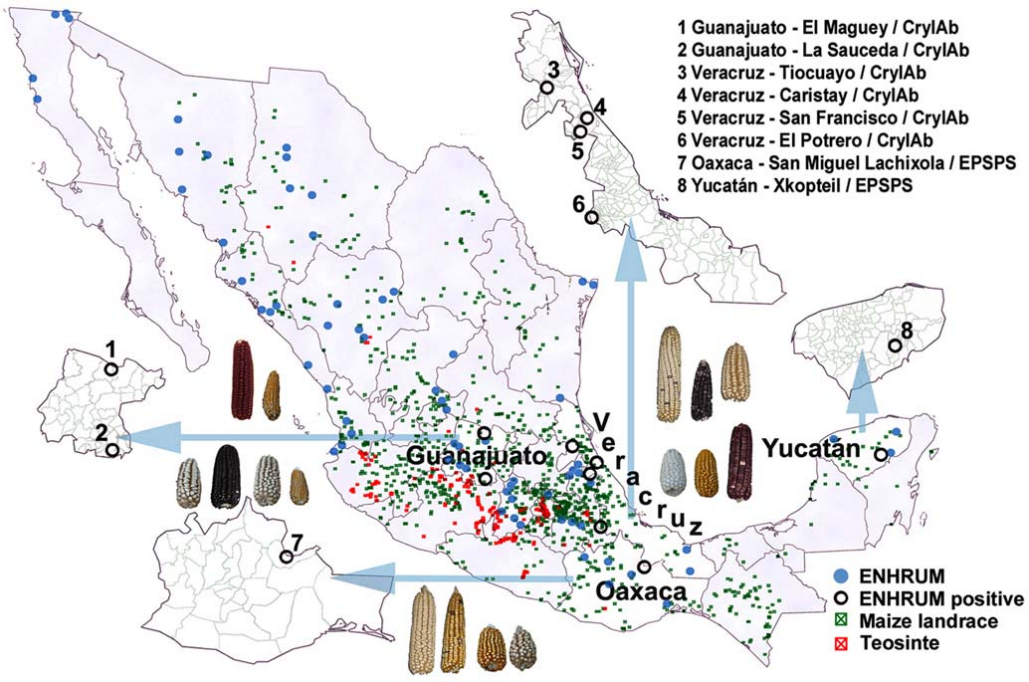
Distribution of survey sample and maize populations. ENHRUM localities (blue), including those where transgenic proteins were detected (black circles). Distribution of teosinte (red) and maize landrace (green) according to INIFAP and CIMMYT genebank collections. Geographic data provided by ENHRUM and Campo Experimental Valle de México, Instituto Nacional de Investigaciones Forestales y Agropecuarias (INIFAP) were processed with ArcInfo.

Immunoassays used to monitor for the activity of recombinant proteins in the collection yielded 6 positive samples for CP4/EPSPS and 10 for Cry1Ab/Ac, representing 1.8 and 3.1% of seed lots nationwide, respectively (Table 4). CP4/EPSPS was present only in the southeast region. Within this region, it was most common in the state of Oaxaca (P = 0.01) but was also found in Yucatán (Fig. 1). Cry1Ab/Ac's distribution also was aggregated in the southeast region (P<0.01) but in this case in the state of Veracruz (P = 0.05). It was present in the state of Guanajuato in the west-central region as well. It is noteworthy that 5% of samples nationwide expressed activity of recombinant proteins, and no samples showed activity of both proteins.

**Table 4:** Expression of transgenic proteins in Mexican maize seed lots in 2002.

Region	ELISA for CP4/EPSPS		ELISA for Cry1Ab/Ac	
	No. of seed lots	Percent of positives^1^	No. of seed lots	Percent of positives^1^
National	327	1.83 (0.76–3.77)	321	3.12 (1.60–5.45)
Southeast	108	5.56 (2.28–10.99)	105	7.62 (3.56–13.70)
Center	142	0.00 (0.00–1.34)	139	0.00 (0.00–1.37)
West-Center	68	0.00 (0.00–2.79)	68	2.94 (5.55–9.12)
North	9	0.00 (0.00–19.22)	9	0.00 (0.00–19.22)

1 Confidence intervals (in parentheses) were estimated using profile-likelihood and binomial ln(-ln) transformations.

All positive-testing samples whose type and source were identified were landraces obtained through informal seed systems. Farmers had obtained 55% of these seed lots prior to 1996, mostly locally. They had mixed 15% of them with other seed before or during 2002, and diffused 38% across farms during the last 5 years, 3.0 times (*C*) on average. This is not significantly different from diffusion rates for landrace seed lots in general, 41% of which were diffused an average of 3.2 times during the same period. In a locality in Veracruz, four out of ten seed lots of the *chipahuac* variety expressed Cry1Ab/Ac, but twenty seed lots of other landraces did not. Since pollen exchange would result in a more even dispersal of transgenes across landraces, the previous pattern is suggestive of dispersal through seed diffusion.

## Discussion

Our results suggest that 5.0% of seed lots in Mexican maize seed stocks could express recombinant proteins despite the moratorium on GMV plantings. All seed lots testing positive were landraces; *i.e.*, no GMV seed lots were found in the sample. Even allowing for sample error, transgenic seed lots were at least 10 times more abundant in seed stocks than GMV lots, since the observed frequency of transgenic seed lots is 5.0%, while the upper limit of the confidence interval of GMV frequency is 0.5%. If we were to explain this ratio as the result of pollen exchange and natural selection alone, it would imply a remarkably strong reproductive advantage for GMVs. Out of every field sown to a GMV, pollen would have spread to more than 10 fields in amounts sufficient to reach detectable frequencies given our sampling protocol (>4.5%). However, it seems unlikely that transgenes in commercial maize GMVs (*e.g.*, Bt or glyphosate-resistant maize) can confer such advantage in Mexico. Susceptibility to Cry toxins varies across insect species as well as within species [42]. Cry toxins expressed by Bt maize lines in 2002 target the European corn borer (*Ostrinia nubilalis*), which is not a pest in Mexico. In contrast, some locally important insect pests—*e.g.*, the fall armyworm (*Spodoptera frugiperda*)—are significantly less susceptible to these toxins [42], [43]. There are no reports on the efficacy of Bt maize against other major pests in Mexico, *e.g.*, the maize weevil (*Sitophilus zeamais*). Likewise, there is no selection in favor of plants carrying CP4/EPSPS, which confers tolerance to glyphosate. Glyphosate-based herbicides are rarely used in subsistence maize production and were not reported in localities where CP4/EPSPS was detected. Alternatively, the observed distribution of transgenes might be explained in terms of seed dynamics.

Transgene dispersal requires a combination of the following processes: commercial release of seed of a GMV through formal seed networks; adoption and use of GMV on farm; hybridization of a GMV and a non-transgenic variety (whether a landrace or an improved variety); diffusion of transgenic seed lots through informal seed systems; diffusion of transgenic grain through grain markets; and use of transgenic grain as seed. GMVs expressing Cry1Ab/Ac, such as MON810 or Bt11, might have been imported and sown in Mexico as early as 1997. After hybridizing with a landrace, Cry1Ab/Ac could have dispersed through informal seed systems and local grain markets for up to 5 years before seed in our sample was harvested in 2002. The window for dispersal was much shorter in the case of CP4/EPSPS, whose most likely source is NK603, released in the US in 2001. NK603 seed might have been imported and sown in Mexico in 2001, dispersing for only one year before our sample was collected. Imported grain expressing CP4/EPSPS would have been available by 2002, making hybridization possible but leaving no time for further dispersal.

A high rate of seed replacement might help explain the absence of GMVs in the sample. In Mexico, an estimated 92% of non-local (*i.e.*, introduced) seed is replaced after a single cycle [14]. If only 8% of GMV lots were saved across cycles, following this pattern, GMVs might be 12.5 times more abundant in the fields than in seed stocks. Over 19% of fields in northern Mexico might have been sown to GMVs in 2002 without being detected (Table 4). Pervasive seed replacement limited ENHRUM's collection of seed in that region [14], which includes the states of Tamaulipas and Chihuahua, where use of imported GMV seed has been reported (*e.g.*, Foro sobre la Minuta con Proyecto de Ley de Bioseguridad de Organismos Genéticamente Modificados, Salón Legisladores, Congreso de la Unión, August 6, 2003; Pérez M, Cientos de hectáreas, sembradas de maíz transgénico en Chihuahua. *La Jornada*, October 29, 2007.) However, even if transgenes were present and dispersed across fields via pollen, a high seed replacement rate would have prevented their survival across cycles. Overall, it is not surprising that transgenes were not found in northern Mexico even if they were present.

In principle, high transgene frequencies in other regions could be due to a high migration rate [44] through repeated introductions of GMV seed. But, foreign seed introductions are relatively rare outside northern Mexico (Table 1), and a low seed-diffusion rate in that region would curtail transgene dispersal through informal seed systems into other regions. A more widespread route for transgene dispersal would be grain markets. Seed acquired as grain (*i.e.*, “grain seed”) is not often saved; it is replaced 4 times more often than seed acquired from neighbors (Tables 2 & 3). In 2002, grain seed bought in Diconsa (a presumed source of transgenes) or seed acquired in the US might have been sown in up to 1.8% of fields in west-central Mexico (Table 1), but none of it was saved into 2003 by the surveyed farmers. Some of this germplasm might have made its way into local seed stocks nevertheless.

Unlike commercial hybrid seed, which is replaced methodically, the most likely reason for replacing grain seed is bad performance. Although local grain might perform well as seed, grain seed of improved varieties, including GMVs, is not likely to perform well because it has already been subjected to one generation of inbreeding even prior to sale. It is possible, therefore, that farmers usually find non-local grain seed inappropriate and discard it. Still, some grain seed is occasionally perceived as a source of valuable traits and backcrossed into local varieties. Improved seed often is crossed with local seed to adapt the former to local conditions or impart specific traits to the latter [6], [14], [21]. Commercial hybrids can loose vigor rapidly, but farmers diffuse seed fast and cross it promptly [14]. This could also be the case of grain used as seed, which diffuses well but disappears unusually fast (Tables 2 & 3). Hence, GMV grain seed might have disappeared as a distinct seed type (and genotype) while its genes remained within the gene pool. Recombinant traits in commercially available maize GMVs may have no evident advantage in Mexico, but it is not necessarily these traits that farmers might have perceived as valuable and backcrossed into local maize, especially if GMVs are not phenotypically distinct from their hybrid isolines. Thus, intentional mixing of seeds might help explain both the rarity of GMVs in seed stocks relative to transgenic landrace seed lots and the apparent high frequency (>4.5%) of individual transgenic seeds within the latter.

Overall, seed management could have led to the transfer of transgenes from various sources into landraces and their dispersal within west-central Mexico, where the introduction and diffusion of improved seed through informal systems are highest [14]. Yet, it is hard to explain the abundance of transgenes in the southeast, where use of foreign seed or Diconsa grain seed is the lowest (Table 1). Grain smuggling and grain brought from northern Mexico might increase the possible sources of transgenes in the southeast, but these sources cannot account for the region's estimated 13.2% of transgenic seed lots (Table 4). Although genes can disperse remarkably fast via seed, the implicit rate of seed diffusion is well in excess of 10-fold—exceedingly high by current standards. Valuable new seed lots are propagated rapidly—an average of 6.6 times in five years [14]—but <0.7% of all seed lots in ENHRUM diffused >10-fold in the 5 years prior to the survey. Moreover, all potential sources of transgenes, including introduced seed and grain seed, exhibit high replacement rates but low diffusion (Table 2), so we would expect their populations to decline in numbers within a locality rather than spreading. Also, since cultural and environmental heterogeneity limits the diffusion of seed across localities [14], [17], transgenes would have to disperse autonomously in every locality. Accidental transfer of transgenes across fields might also be limited in the southeast, since seed of improved varieties (including GMV grain seed) often is ill-adapted to conditions in the region, bound to pollinate asynchronously, produce less pollen and yield poorly [45].

In sum, the frequency of transgenes in southeast Mexico is not consistent with i) the current use of germplasm from presumed sources of transgenes or ii) the rate at which germplasm normally spreads through informal seed systems even under the most favorable conditions. Observed frequencies suggest that either additional sources of transgenes were available in the past or seed from available sources was diffused more extensively. One possibility is that transgenes were diffused through the formal seed system, particularly by local seed companies targeting sub-prime agricultural areas. During the nineties, INIFAP, Mexico's leading agricultural research institution, promoted non-conventional maize hybrids—*i.e.*, a cross of a local variety and a hybrid—as an option for these areas, where registered varieties are not competitive [46]. Development and release of genetically modified materials is regulated by law and has not been reported. Certified seed must meet origin, genetic identity and quality standards. However, only a fraction of commercial seed in Mexico is certified, and sale of non-certified seed (including non-conventional hybrids) is not regulated. Transgenes might accidentally find their way into non-certified seed through various sources and mechanisms, as they have done in the US [47]. In Mexico, their source could be the seed of a GMV grown locally or of a local variety that has already been introgressed with exotic germplasm originating in the US.

While these scenarios are clearly more likely for GMVs released in 1997 than for those released in 2001, none of them are highly probable under current conditions. Formal seed systems are usually limited outside prime agricultural areas by a lack of demand for improved varieties. Seed obtained from the seed industry accounted for only 0.8% of the southeast's total in 2002. Nevertheless, the reach of formal systems into sub-prime areas was much greater in the recent past. Government programs such as *Kilo por Kilo*, which operated between 1996 and 2001, extended their reach significantly [48]. Although *Kilo por Kilo*'s express goal was to promote the use of certified seed in prime areas, it extended into sub-prime areas where it distributed non-certified seed, often ill-suited to local conditions [48], [49]. In 2001, most seed distributed through the program failed to meet federal standards, prompting auditors to recommend “a more strict record” of the origin and sanitary standards of seed [48].

Visible signs of government intervention on local seed stocks can dissipate fairly quickly. In 2002, only 0.4% of maize seed lots sown by rural farmers were reported as having a governmental source (Table 1). Yet, widespread diffusion of improved varieties can have a lasting influence on local germplasm [21]. All three lots from a governmental source in the ENHRUM collection were acquired by their respective farmers in 2001; one was identified as an improved variety, another as a landrace, and a third reportedly had mixed origins. Samples of the last two, collected in west-central Mexico, tested negative. Alternative explanations to transgene dispersal in southeastern Mexico should be explored, including containment failures of NK603 prior to its release or of events not released commercially, which has occurred in the United States before.

It is of interest whether transgenes will disappear or continue to disperse across the Mexican landscape. It is likely that GMVs brought into cultivation have been discarded, but some of them might have been incorporated by farmers into local seed stocks. Such materials are usually managed indistinguishably from local seed, which might prevent their disappearance wherever maize populations are relatively closed and stable, as in the southeast highlands [14], [16], [17]. Notably, there are no evident differences in the diffusion rates of positive samples and other landrace seed lots in the survey. In contrast, in areas where seed populations are constantly infused by improved seed and grain, as in west-central Mexico, existent transgenic seed lots could disappear gradually as local stocks are replaced; but exotic hybrids, including GMVs, might be introduced anew for the same reason.

Although transgene flows within crop fields are relatively well understood, analysis of highly-structured crop populations still poses serious challenges [12], [13], [20]. Studies seeking to estimate transgene frequencies in centers of crop diversity must deal with significant scale issues [13]. Spatial aggregation of transgenes facilitates their detection within particular populations but lowers the overall probability of detection across populations [12]. In order to design an efficient sampling framework, some prior knowledge of the distribution of allele frequencies is needed [12], [13]. Studies to date show that transgenes can be extremely rare in some localities even when neighboring populations exhibit relatively high frequencies [9]–[12]. Yet, little is known about the distribution of transgenes at larger scales. Our estimates of the frequency of transgenic seed lots across maize populations in Mexico should provide guidance to future studies. Although their distribution continues to be aggregated, transgenes seem to be more widely spread than previously thought [9]–[12].

Understanding transgene dynamics within crop metapopulations poses a different set of challenges. Some have speculated that transgene dispersal is unsurprising and inevitable [26]. Hypotheses on the disappearance of transgenes from landrace populations are even more controversial [9], [10], [12], [13]. Including this report, there is now evidence of transgenes in Oaxaca in 2001, 2002 and 2004 but no indication of whether this is the result of dispersal across cycles and localities or of repeated introductions [12]. Crop populations are subject to evolutionary forces operating at different spatial and temporal scales [6], [14]–[17], [30]. Analyzing the implications of seed dynamics on population genetics requires resolving conceptual and methodological differences between the disciplines that traditionally study these forces. Analysis of transgene dynamics and frequencies in crop fields and seed stocks serves different purposes. Unlike natural forces operating in the field, management of seed stocks determines the survival of entire populations, often irrespective of their fitness advantage [14], [17]. Our analysis of these forces suggests that the potential for transgene survival and dispersal through informal seed systems varies widely among and within regions. Informal systems provide only weak linkages between seed stocks across regions. Grain markets and formal seed systems can tighten these linkages; yet, little is known about how these channels are linked.

Regulation on the release of genetically modified crops in many developing countries is pending. In Mexico, current law initiatives assume that the spread of transgenes into centers of crop origin and diversification can be either prevented or reversed if commercial release of GMVs is restricted to areas of industrialized agriculture. Our results show that this approach might be ineffective. While screening protocols for commodity stocks and imports have improved [27], [39], tracking grain flows within Mexico is a daunting task posing formidable challenges. Explaining the precise circumstances surrounding containment failures in the US has proved difficult [47], [50]. It is even more difficult in Mexico, particularly after deregulation of the seed industry in 1991. Deregulation allowed the industry to sell non-certified seed and abolished the requirement of keeping or depositing samples with the government's official genebank. Many small seed companies operating during the nineties have disappeared, leaving few records. At the same time, deposits in the official genebank consist of 400 seeds from an unspecified number of ears, which might exclude genetic variation in landraces and their crosses, such as non-conventional hybrids. Under these conditions, only high transgene frequencies can be detected with confidence [12], [13].

In order to fully assess the potential for transgene dispersal in centers of crop origin and diversification, further research is needed on germplasm flows through formal and informal seed systems and grain markets, and on the interactions between these channels.
